# Effect of Breastfeeding on the Course of Respiratory Syncytial Virus Infection in Infants: A Single-Center Retrospective Study

**DOI:** 10.3390/pediatric17050110

**Published:** 2025-10-21

**Authors:** Anna Maćkowska, Jakub Nowicki, Elżbieta Jakubowska-Pietkiewicz

**Affiliations:** Department of Pediatrics, Newborn Pathology and Bone Metabolic Diseases, University of Lodz, 91-738 Lodz, Poland; jakub.nowicki@umed.lodz.pl (J.N.); elzbieta.jakubowska-pietkiewicz@umed.lodz.pl (E.J.-P.)

**Keywords:** breastfeeding, RSV, feeding, infants, respiratory syncytial virus

## Abstract

**Background/Objectives**: Respiratory syncytial virus (RSV) is one of the main pathogens causing infections of the respiratory system. In our study, we investigated whether breastfeeding, compared to feeding with formula milk, has an effect on RSV infection in newborns and infants. **Methods**: We analyzed 51 patients hospitalized at Department of Pediatrics, Newborn Pathology and Bone Metabolic Diseases, University of Lodz, with RSV infection. The infants were divided into two groups by the type of the feeding mode: breast milk or formula milk. **Results**: Breastfed infants were hospitalized for less time compared to those fed with milk formula (8 days vs. 11 days, *p* < 0.05). A multivariate linear regression model showed that babies fed with formula milk spent an average of 1.7 days longer in hospital than those fed with breast milk (95% Cl 0.247–3.209). **Conclusions**: Breastfeeding could reduce the risk, and in some cases, also the severity of RSV infection.

## 1. Introduction

Respiratory syncytial virus (RSV) is one of the main pathogens causing infections of the respiratory system. In children, especially those under the age of 5 years, RSV leads to infections of the upper and lower respiratory tract, mainly bronchiolitis, but also pneumonia. The course of the infection can be acute and lead to respiratory failure. It is estimated that RSV is the most common cause of hospitalization in young children with respiratory infections. Every year, RSV accounts for approximately 120,000 deaths in children under the age of five years [1]. Children under 6 months of age are a particularly large group [2]. The overall rate of hospitalization for lower respiratory tract infections due to RSV is 0.5–4%. It has been shown that RSV infection in early life may be associated with the development of recurrent episodes of wheezing and asthma later in life [3]. The highest risk of severe RSV infection occurs among premature infants, children with comorbidities, particularly respiratory diseases, such as bronchopulmonary dysplasia, heart defects, and Down syndrome [4]. The identified risk factors also include low birth weight, maternal smoking during pregnancy, nursery/kindergarten attendance, poor parental education, multiple pregnancies, delivery during winter months, exposure to tobacco smoke, positive history of atopy and lack of breastfeeding [5]. In adults, the infection usually has a mild, self-limiting course, while those at risk for a severe course are individuals aged over 65 years and patients with chronic obstructive pulmonary disease [6]. An increase in the incidence of RSV infection is observed seasonally, i.e., in winter and early spring [7]. In Europe, there occurs a moderate correlation between the incidence of RSV and latitude, i.e., the higher the latitude, the greater the number of cases [8].

Breastfeeding is known for many health benefits. One of them is to be a protective factor against both upper and lower respiratory tract infections, including RSV infection. In our study, we investigated whether breastfeeding, compared to feeding with formula milk, has an effect on RSV infection in newborns and infants.

## 2. Materials and Methods

### 2.1. Participants and Measures

We retrospectively analyzed the medical records of patients with RSV infection confirmed by antigen tests who had been fed with either breast milk or milk formula and were hospitalized between August 2021 and February 2022 at the Department of Pediatrics, Neonatal Pathology and Metabolic Bone Diseases of the Medical University of Lodz. Information was obtained on the patients’ sex and age, their perinatal history, weight on admission and discharge, feeding regimen, CRP levels on admission, treatment administered and length of hospitalization.

The material for laboratory tests was collected by well-qualified nursing staff. RSV infection was confirmed using Abbott’s BINAX NOW RSV Antigen Test (sensitivity: 94%, specificity: 100%), while CRP concentration was determined using the Abbott Alinity C analyzer. The reference range for CRP concentration was <5 mg/dL. Analyses were performed at the laboratory of the University Center for Pediatrics of the Central Clinical Hospital of the Medical University of Lodz. Body weight in the infants was measured using a RADWAG C315.D scale.

Fifty-three patients with confirmed RSV infection were included in the study. After preliminary analysis of the data, two babies were excluded from the analysis due to outliers in the study group (delivery at 28 weeks gestation, age at admission over 250 days).

The babies were divided into two groups by the type of the feeding mode: breast milk or formula milk. We investigated whether their perinatal history influenced the way the patients were nourished (Table 1), as well as whether the feeding schedule influenced the length of hospitalization, weight gain during hospitalization, levels of inflammatory parameters on admission and the need for antibiotic therapy, oxygen therapy or other forms of treatment (Table 2).

### 2.2. Statistical Analyses

The relationships between the length of hospitalization and the other variables were then analyzed (Table 1 and Table 2).

The distributions of variables were examined by analyzing histograms and the result of the Shapiro–Wilk test. Qualitative variables were described by frequencies and percentages of occurrence, and their analysis was performed based on the following tests: Chi2, Fisher and Fisher–Freeman–Halton. Continuous variables were described using the median and the first and third quartiles. Subsequently, they were analyzed using the U-Mann–Whitney test. Analysis of variance was performed using the Kruskal–Wallis test, and the relationship between the variables was tested by calculating the Spearman rank correlation coefficient. Factors that showed a statistically significant effect on the length of hospitalization in the above statistical analyses were included in univariate linear regression models. Variables that demonstrated a significant association with the length of hospitalization in univariate models were included in the multivariate linear regression model. Analyses were performed using IBM SPSS Statistics ver. 28.0 software (IBM Co., Armonk, NY, USA). *p* < 0.05 was considered as statistically significant.

## 3. Results

Among the 51 patients analyzed, 52.9% were girls and 47.1% boys. Slightly over 47% of the patients were fed with mother’s milk (*n* = 24). None of the infant’s mothers had been vaccinated against RSV. Some differences were found between the groups of patients fed with breast milk and those given formula. Babies breastfed were statistically significantly more often born by spontaneous delivery (*p* < 0.01), at a later week of gestation (39 vs. 37, *p* < 0.001) and had a higher birth weight (3540 g vs. 3200 g, *p* < 0.01). These patients were hospitalized at an earlier postnatal age (21 days vs. 40 days of life, *p* < 0.01), while the hospitalization itself lasted shorter (8 days vs. 11 days, *p* < 0.05) (Table 1 and Table 2).

Analyses of the relationships between the length of hospitalization and other variables show that the time spent in the hospital was determined by the need to include antibiotic therapy and oxygen therapy, the use of hypertonic saline nebulization (3% NaCl) in the treatment, and the way the infants were fed. Those who required antibiotic therapy and oxygen therapy spent more time in hospital (11 days vs. 9 days, *p* < 0.05 and 11 days vs. 8 days, *p* = 0.01, respectively). Children treated with 3% NaCl nebulization during their hospital stay required a shorter hospitalization period than those who did not receive this agent (10 days vs. 15 days, *p* < 0.05). Similarly, shorter hospitalization time was observed in breastfed neonates and infants as compared to those fed with milk formula (8 days vs. 11 days, *p* < 0.05) (Table 1 and Table 2).

Further analysis showed that the length of hospitalization correlated negatively with the week of gestation in which the baby was born; however, the correlation was weak (r = −0.29, *p* < 0.05). There were no statistically significant correlations between the length of hospitalization and age, weight or CRP levels at hospital admission.

Based on univariate and multivariate linear regression models, factors associated with the length of hospitalization of newborns and infants with RSV infection were identified (Table 3).

In this regression models, it was observed that the week of gestation at which the child was born was not significantly related to the length of hospitalization (B = −0.41, 95% Cl −0.85–0.04). The other explanatory variables were significantly related to the length of the child’s hospitalization and were included in a multivariate linear regression model. The model created was statistically significant (*p* < 0.001) (Table 3).

A multivariate linear regression model showed that after standardizing for all the explanatory variables included in the model, the use of antibiotic therapy was not significantly related to the length of hospitalization (B = 1.13, 95% Cl −0.469–2.726). In children who were treated with nebulization with 3% NaCl, the mean length of hospitalization was shorter by about 2.5 days on average (95% Cl −5.028–−0.069). The need for oxygen therapy increased the duration of hospitalization by an average of 1.9 days (95% Cl 0.300–3.530). Babies fed with formula milk spent an average of 1.7 days longer in hospital than those fed with breast milk (95% Cl 0.247–3.209) (Table 3).

## 4. Discussion

RSV infections are common problems in everyday paediatric practice. For this reason, methods of preventing this infection are being researched. Currently, immunoprophylaxis of the infection is provided in Poland in the form of a monoclonal antibody, palivizumab. The supply is reserved for infants at high risk of severe infection and, due to the short duration of protection, the vaccine is administered before and during the RSV infection season [9]. In Poland, immunoprophylaxis covers children up to 12 months of age born ≤28 weeks of gestation or with bronchopulmonary dysplasia, as well as children up to 6 months of age born at 29–32 weeks of gestation.

Another monoclonal antibody, nirsevimab, is approved by the EMA and used in many countries in Europe for the prevention of RSV infection. It shows greater neutralizing potential and a longer half-life, so it can be used as a single dose. Safety and efficacy in preventing RSV infection in newborns and infants born ≥29 weeks of gestation has been confirmed in clinical trials [10,11,12].

A meta-analysis of 27 studies using nirvesimab in children up to 12 months of age showed a significant reduction in the risk of hospitalization for RSV infection (OR 0.17; 95% CI 0.12–0.23), intensive care unit (ICU) admission (OR 0.19; 0.2–0.29) and lower risk of lower respiratory tract infection due to RSV infection (OR 0.25; 0.19–0.33) [13]. In many European countries such as Spain, France, Switzerland, Luxemburg, Belgium and Germany, passive immunization with nirsevimab has already been introduced in universal vaccination programs. Currently, there are no recommendations for specialized therapy, only symptomatic treatment and, in severe cases, respiratory support are used.

In August 2023, the European Commission issued a decision to authorize the approval of a vaccine for pregnant women for passive protection against lower respiratory tract diseases caused by respiratory syncytial virus in infants from birth to 6 months of age following maternal vaccination during pregnancy. Abrysvo (Pfizer) in its formulation contains F antigens (F glycoprotein) of RSV subgroups A and B, produced by recombinant DNA in Chinese hamster ovary cells. The F protein in the prefusion conformation is the main target of neutralizing antibodies that block RSV infection. When administered intramuscularly, F antigens in the prefusion conformation induce an immune response that protects against RSV-induced lower respiratory tract disease. The MATISSE clinical trial proved the efficacy of this vaccine in pregnant women. The efficacy in preventing severe, confirmed RSV-induced lower respiratory tract infection at 90 and 180 days after birth was 81.8% and 69.4%, respectively. Side effects were mild and mostly included injection site pain, headache and muscle pain. It is recommended that one dose of the vaccine be administered between 24 and 36 weeks of pregnancy [14,15,16].

The World Health Organization (WHO), as well as numerous scientific societies such as the American Academy of Pediatrics (AAP) and the European Society for Pediatric Gastroenterology, Hepatology and Nutrition (ESPGHAN), list numerous health benefits of breastfeeding, such as reduced incidence of infectious diarrhea, otitis media, bacterial meningitis, respiratory tract infections, necrotizing enterocolitis, urinary tract infections, lower risk of overweight, obesity, asthma, diabetes, Crohn’s disease, lymphoid and myeloid leukemia and others. For this reason, the abovementioned organizations recommend exclusive breastfeeding for a minimum of 6 months. The risk of hospitalization due to lower respiratory tract infections in children aged less than 6 months has been shown to decrease by 72% in infants exclusively breastfed for a minimum of 4 months [17].

Human milk contains specific immunoregulatory and immunomodulatory factors that can promote maturation of the infant’s immune competence. In breast milk, there are substances responsible for protection against infections, such as lactoperoxidase, lysozyme, lactoferrin, catalase, superoxide dismutase, glutathione peroxidase, secretory immunoglobulins (SIgA), immunoglobulins IgM, IgG, IgD, interleukins, especially IL-4, IL-10, IL-12, IL-18, growth factors, i.e., epidermal growth factor (EGF), insulin-like growth factor-I (IGF-1), nerve growth factor (NGF), transforming growth factor α and β (TGF α and β) [18].

The beneficial effect of breastfeeding on the incidence of respiratory tract infections has been widely reported in the literature. Many of the studies also included RSV infections. As mentioned above, there are factors in human milk that regulate the immune response in infants. One of them is lactoferrin. It has been shown that the concentration of lactoferrin in human milk is significantly (up to 1000 times) higher as compared to plasma concentration. Lactoferrin, by chelating iron compounds that are necessary for microorganisms to multiply, limits their growth. Additionally, it is postulated that lactoferrin reduces the formation of bacterial biofilms. It also shows immunomodulatory and immunotropic effects [19]. Human milk oligosaccharides (hMOSs) seem to be one possible protective mechanism. In a review by Tonon et al. they were shown to reduce viral load and influence inflammatory signaling in RSV infection [20]. hMOSs are also a source of energy for the microbiota found in the intestines, which stimulates the growth of Bifidobacterium, Lactobacillus and Bacteroides bacteria in the child’s digestive tract. These bacteria also appear to play an important role in preventing respiratory infections. Children fed with formula milk have a different microbiota composition, which may also affect their immunity. Attempts are being made to add probiotics to formula milk, but it is almost impossible to recreate the microbiota found in breast milk [21].

In a study by Dornolles et al., a longer duration of exclusive breastfeeding was associated with shorter hospital stays due to RSV infection and a decreased need for passive oxygen therapy. Breastfeeding continued for less than a month increased the incidence of RSV infections. Also, breastfed infants were less likely to require hospitalization than those who were not breastfed [22].

A retrospective study by Jiménez-Nogueira et al. analyzed the effect of breastfeeding on the course of RSV infection in children under 6 months. The analysis showed that even brief breastfeeding of at least 15 days or more reduced the risk of hospitalization in the intensive care unit and mechanical ventilation. Furthermore, not breastfeeding for a minimum of one month was a risk factor for admission to the ICU, and not breastfeeding for two months was predictive of the need for mechanical ventilation [23]. A systematic review analysing the impact of breastfeeding on the course of RSV infection in children also confirmed positive effects. The study included 19 articles from 31 countries, covering a total of 16,787 children under 12 months of age. It was shown that breastfeeding for 4–6 months reduced the number of hospitalisations, the length of hospitalisation, the need for oxygen supply and the risk of admission to intensive care unit [24].

A multicenter, retrospective, Korean study conducted by Jeong Jang et al. analyzed hospitalization data from 411 children under the age of 1 year, admitted to four pediatric centers for RSV infection (pediatric and intensive care units). The children were divided into groups according to the mode of feeding—breast (BMF), artificial (AMF) or mixed (MF). The prevalence of oxygen therapy use was significantly lower in the breast-fed group, than in the artificially fed group and in the mixed group (4.3%, 13.5% and 8.1%, respectively; *p* = 0.042). The odds ratio (OR) for the use of oxygen therapy was higher in the AMF group than in the BMF (adjusted OR, 3.807; 95% confidence interval, 1.22–11.90; *p* = 0.021). Among the breastfed infants, the rate of admission to the intensive care unit was 1.1%, which was lower compared to the MF (3.5%) and AMF (4.5%) groups; however, the difference was not statistically significant (*p* = 0.338) [25]. In our study, there were no differences in the frequency of oxygen therapy use depending on the feeding method. We showed that the required hospitalization period in breastfed children was on average 1.7 days shorter than among artificially fed children (95% Cl: 0.247–3.209).

In the study, we also showed that babies who were breastfed were more likely to be born by spontaneous delivery, and closer to their due date. Our findings are consistent with those reported in the literature. Numerous papers emphasize the impact of uninterrupted skin-to-skin (STS) contact after birth and early initiation of lactation during this contact. A randomized study by Srivastava et al. showed that newborns who had early STS contact following spontaneous delivery had fewer suckling problems and were more likely to be exclusively breastfed [26]. The duration and occurrence of STS contact is influenced by the way of delivery. Among women undergoing surgical deliveries, this option is considerably limited. However, STS contact should also be provided after cesarean section. A study by Guala et al. showed that skin-to-skin contact with the mother after cesarean section increased the percentage of women exclusively breastfeeding at hospital discharge and at 3 and 6 months as compared to STS contact with the father and not providing this option [27]. Decisions to perform a cesarean section should be made with caution and after careful consideration of all indications, as well as the potential risks of the decision.

Guidelines on the treatment of bronchiolitis clearly emphasize that the use of antibiotic therapy, inhaled or systemic corticosteroids and broncholytics does not affect the duration of the disease, severity of symptoms or later prognosis. It is emphasized that in the case of hypoxemia, maintaining the child’s proper hydration and the use of oxygen therapy are of key importance. Infants with RSV infection should always be assessed for dehydration which is much more likely to occur as a result of tachypnea, production of large amounts of airway secretions, difficulty sucking and decreased appetite. Whenever possible, infants should be fed by the oral route, i.e., breast, breast milk or formula milk from a bottle, or with the use of an intragastric probe if sucking is difficult. They may also require intravenous hydration or parenteral nutrition [28,29,30].

Children with RSV infection may develop hypoxemia due to bronchial obstruction and an imbalance between ventilation and perfusion. The saturation values at which passive oxygen therapy should be implemented remain a contentious issue; however, in most cases they are recommended to be below 90–92% [28,29,30]. In our study, patients who received passive oxygen therapy were hospitalized longer by about 1.9 days. This may be due to the severity of the disease course since patients with more serious symptoms needed oxygen supplementation. In a study by Nishimura et al. conducted among 203 infants with RSV infection, a multivariate linear regression model showed that children who were exclusively breastfed were less likely to require oxygen therapy [31].

NICE guidelines do not recommend the routine use of hypertonic saline solutions by nebulization [30]. In our study, we demonstrated that the hospitalization period among children who received such treatment was shorter (10 days vs. 15 days, *p* < 0.05). It is possible that this effect was achieved by reducing nasal congestion, which may shorten the incubation period of the virus and facilitate the expectoration of retained secretions. A multicenter randomized clinical trial conducted by Morikawa et al. among 128 children with RSV infection showed no effect on the length of hospitalization, with good tolerance of such treatment [32]. A study by Faber et al. showed no effect on clinical assessment and no pathological lung sounds in RSV infection patients treated with hypertonic saline [33]. In contrast, another study by Stobbelaar et al., analyzing 104 children hospitalized in intensive care units due to RSV infection, revealed that patients receiving hypertonic saline solutions by nebulization required shorter periods of hospitalization and respiratory support [34].

Our study has certain limitations, which mainly result from its retrospective nature. Another limitation is the small sample size. Moreover, there were no children in our group who received RSV prophylaxis (antibodies or maternal vaccination)—including such children in the analysis could bring further benefits. More data on this topic, particularly observational studies is required, to increase the percentage of breastfeeding mothers. Additionally, more research adjusted for the severity of RSV infection is needed to determine whether breastfeeding status affects the outcome of hospitalization (length of stay).

## 5. Conclusions

Currently, treatment of RSV infection is based solely on symptomatic treatment. As shown in our study and in the literature data, breastfeeding could reduce the risk and, in some cases, the severity of RSV infection too. Breast milk contains neutralizing antibodies and other anti-infective components, as well as substances stimulating the infant’s immune system. For this reason, we should encourage women to breastfeed by promoting many benefits of this natural method, thus also reducing the severity and risk of potential complications resulting from RSV infections. Children with weakened immune systems or premature babies, who are more susceptible to infections, can particularly benefit from this.

## Figures and Tables

**Table 1:** Patients’ baseline characteristics. UMW—U-Mann–Whitney test, K-W—Kruskal–Wallis test, FFH—Fisher–Freeman–Halton test, Chi2—Chi2 test.

	Total *n* = 51	Type of Feeding			Length of Hospitalization (Days)	
		Breast Milk *n* = 24	Formula Milk *n* = 27	*p*		*p*
**Sex**				0.5 ^Chi2^		0.9 ^UMW^
Girl	27 (52.9)	14 (58.3)	13 (48.1)		10 (8–13)	
Boy	24 (47.1)	10 (41.7)	14 (51.9)		10 (8–13)	
**Pregnancy**				0.1 ^FFH^		0.2 ^K-W^
1	8 (15.7)	2 (8.3)	6 (22.2)		11 (9–14)	
2	32 (62.7)	13 (54.2)	17 (63)		9 (7–12)	
>2	11 (21.6)	9 (37.5)	4 (14.8)		12 (8–13)	
**Delivery**				0.3 ^FFH^		0.4 ^K-W^
1	8 (15.7)	2 (8.3)	6 (22.2)		11 (9–14)	
2	33 (62.7)	15 (62.5)	17 (63)		9 (7–12)	
>2	11 (21.6)	7 (29.2)	4 (14.8)		11 (8–13)	
**Type of delivery**				**<0.01** ^Chi2^		0.9 ^UMW^
natural childbirth	20 (44.4)	13 (68.4)	7 (26.9)		9 (8–13)	
cesarean section	25 (55.6)	6 (31.6)	19 (73.1)		11 (7–12)	
**Week of pregnancy**	39	39	37	**<0.001** ^UMW^	-	
	(37–39)	(39–40)	(35–39)			
No data	1		1			
**Birth weight [g]**	3400	3540	3200	**<0.01** ^UMW^	-	
	(2895–3642)	(3250–3780)	(2680–3580)			
No data	1	1				
**Apgar score**				0.5 ^FFH^		0.14 ^K-W^
9–10	43 (84.3)	22 (91.7)	21 (77.8)		9 (7–12)	
7–8	5 (9.8)	1 (4.2)	4 (14.8)		13 (10–15)	
6	3 (5.9)	1 (4.2)	2 (7.4)		10	

**Table 2:** Patients’ clinical outcomes. Data are presented as frequencies and percentages (%), medians and quartiles (Q1–Q3). UMW—U-Mann–Whitney test, K-W—Kruskal–Wallis test, F—Fisher’s exact test, Chi2—Chi2 test.

	Total *n* = 51	Type of Feeding			Length of Hospitalization (Days)	
		Breast Milk *n* = 24	Formula Milk *n* = 27	*p*-Value		*p*-Value
**Age on admission [days]**	25 (19–47)	21 (15–26)	40 (24–91)	**<0.01** ^UMW^	-	
**Body weight on the day of admission [g]**	4180 (3620–4900)	4155 (3650–4580)	4240 (3620–5620)	0.5 ^UMW^	-	
**Length of hospitalization (days)**	10 (8–13)	8 (7–12)	11 (9–13)	**<0.05** ^UMW^	-	
**Weight gain [g/day]**	25.7 (15–38.8)	27.9 (12.5–38.9)	25 (15–36.6)	0.6 ^UMW^	-	
**CRP [mg/L]**	0.8 (0.3–3.2)	1.2 (0.3–6.2)	0.7 (0.3–2.5)	0.2 ^UMW^	-	
**Antibiotic therapy**				1.0 ^Chi2^		**<0.05** ^UMW^
Yes	30 (58.8)	14 (58.3)	16 (59.3)		11 (9–14)	
No	21 (41.2)	10 (41.7)	11 (40.7)		9 (7–11)	
**Fenoterol + ipratropium**				1.0 ^Chi2^		0.6 ^UMW^
Yes	40 (78.4)	19 (79.2)	21 (77.8)		10 (8–13)	
No	11 (21.6)	5 (20.8)	6 (22.2)		9 (7–14)	
**3% NaCl nebulization**				1.00 ^F^		**<0.05** ^UMW^
Yes	45 (90.0)	22 (91.7)	23 (85.2)		10 (7–12)	
No	5 (10.0)	2 (8.3)	3 (11.1)		15 (10–15)	
**0.9% NaCl nebulization**				0.1 ^Chi2^		0.7 ^UMW^
Yes	33 (64.7)	19 (79.2)	14 (51.9)		9 (8–13)	
No	18 (35.3)	5 (20.8)	13 (48.1)		10 (8–12)	
**Oxygen therapy**				0.6 ^Chi2^		**=0.01** ^UMW^
Yes	31 (60.8)	16 (66.7)	15 (57.7)		11 (9–14)	
No	19 (37.3)	8 (33.3)	11 (42.3)		8 (7–12)	

**Table 3:** One-dimensional and multivariate linear regression models for the length of hospitalization. Multivariate model: R^2^ = 0.28, SE = 2.55, B = 9.90, SEB = 1.44. B–linear regression custom factor, 95% Cl—confidence interval, SE—standard error. R^2^—adjusted.

	Univariate Model			Multivariate Model		
	B	95% Cl	SE	B	95% Cl	SE
**Week of pregnancy**	−0.41	−0.85–0.04	0.22	-		
**Type of feeding**	1.65	0.003–3.294	0.82	1.73	0.247–3.209	0.73
**3% NaCl nebulization**	−3.24	−5.960–−0.517	1.35	−2.55	−5.028–−0.069	1.23
**Antibiotic therapy**	2.01	0.372–3.647	0.81	1.13	−0.469–2.726	0.79
**Oxygen therapy**	2.27	0.614–3.921	0.82	1.91	0.300–3.530	0.80

## Data Availability

The original contributions presented in this study are included in the article. Further inquiries can be directed to the corresponding author(s).
